# Narrative Review of the Use of Genomic-Adjusted Radiation Dose (GARD) in Radiotherapy

**DOI:** 10.3390/cancers17162650

**Published:** 2025-08-14

**Authors:** Jun Yin

**Affiliations:** Department of Biostatistics and Bioinformatics, Moffitt Cancer Center, Tampa, FL 33612, USA; vivien.yin@moffitt.org

**Keywords:** clinical trial, precision medicine, radiotherapy

## Abstract

This narrative review examines the genomic-adjusted radiation dose (GARD), a biologically informed framework developed to personalize radiotherapy by integrating tumor-specific genomic data. GARD combines the radiosensitivity index (RSI), based on gene expression, with the linear quadratic model to estimate patient-specific radiation effect. Since its introduction in 2017, GARD has demonstrated prognostic value across multiple cancer types in retrospective studies. This review summarizes key studies evaluating GARD across various tumor types and clinical contexts. Emerging trials, including a Phase II trial in HPV-positive oropharyngeal cancer, aim to validate GARD-guided dosing in precision radiotherapy. Future efforts may focus on refining RSI, addressing tumor heterogeneity, and validating GARD-guided dosing in prospective settings.

## Simple Summary

This review explains how the genomic-adjusted radiation dose (GARD) can be used to personalize radiation therapy based on a tumor’s genetic makeup. GARD combines gene expression data, through the radiosensitivity index (RSI), with a standard radiation model to predict how well a patient might respond to treatment. Studies have shown that GARD may help guide treatment across different cancer types.

## 1. Introduction

Radiotherapy, while a cornerstone of cancer treatment, faces significant challenges due to intrinsic and acquired resistance mechanisms within tumor cells. Radiotherapy-related anticancer drug resistance is influenced by the radiosensitivity index (RSI), a gene expression-based measure of a tumor’s intrinsic sensitivity to ionizing radiation [1,2,3,4,5]. Tumors with a low RSI are more radiosensitive and tend to respond better to standard radiotherapy doses, while those with a high RSI are more radioresistant and often exhibit suboptimal treatment responses. The variability in RSI across and within tumor types highlights the biological heterogeneity in radiation response, which underlies differential clinical outcomes [6,7]. Consequently, tumors with high RSI are more likely to resist radiotherapy-induced cytotoxicity, contributing to treatment failure and disease recurrence. Incorporating RSI into clinical assessment could help predict and reduce radiotherapy resistance in cancer treatment.

The genomic-adjusted radiation dose (GARD) represents a novel, biologically informed framework designed to personalize radiotherapy by integrating individual tumor genomics into radiation dose planning [8,9,10,11]. Traditional radiotherapy protocols have historically applied uniform doses across patients, neglecting the intrinsic heterogeneity in tumor radiosensitivity. GARD addresses this limitation by combining a gene expression–based radiosensitivity index (RSI) with the linear quadratic model, enabling calculation of a patient-specific estimate of radiotherapeutic effect. By quantifying how effectively a given radiation dose will work based on tumor biology, GARD allows clinicians to tailor radiation prescriptions to maximize therapeutic benefit while potentially minimizing toxicity. This precision-guided approach holds promise for transforming radiation oncology into a more individualized discipline, akin to recent advances in targeted therapies and genomic-driven medical oncology.

The paper is structured as a comprehensive narrative review exploring the development, methodology, and clinical application of the genomic-adjusted radiation dose (GARD) framework in radiotherapy. It begins with an introduction highlighting the limitations of uniform radiation dosing and the role of tumor heterogeneity in treatment response. The methodology section explains how GARD is calculated using the radiosensitivity index (RSI) derived from gene expression and the linear quadratic model. The review then summarizes key retrospective studies that validate GARD across multiple cancer types, presenting clinical outcomes and proposed dose adjustments. A dedicated section describes a prospective Phase II clinical trial implementing GARD-personalized dosing in HPV-positive oropharyngeal cancer. The discussion addresses limitations such as the retrospective nature of most studies, biological and spatial heterogeneity, and the need for further validation. The paper concludes by emphasizing GARD’s promise in guiding biologically informed, personalized radiotherapy, with the potential to improve outcomes and reduce toxicity.

## 2. GARD Calculation

**Figure 1:**
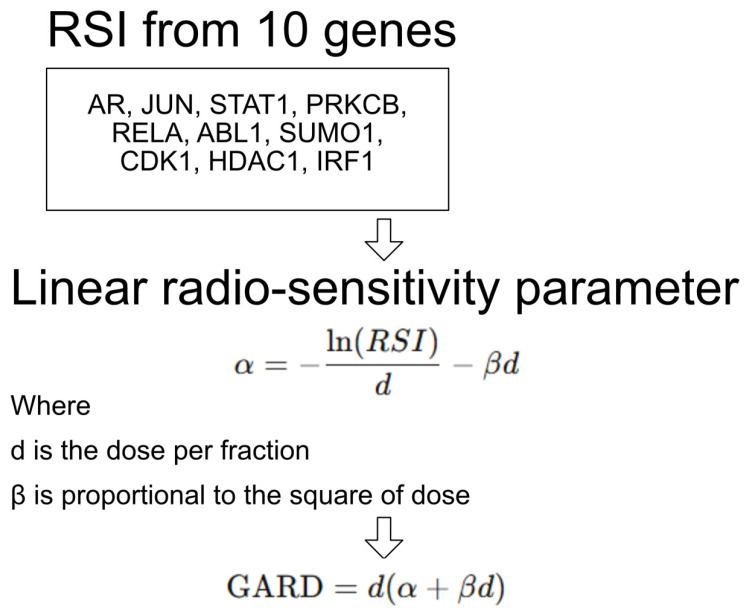
GARD calculation.

GARD is calculated by integrating a patient’s individual radiosensitivity, as measured by a gene-expression-based radiosensitivity index (RSI), with the linear quadratic (LQ) model commonly used in radiobiology [8]. Specifically, GARD quantifies the biological effect of a given radiation dose tailored to the genomic profile of a tumor. The RSI is derived from the expression levels of 10 genes and predicts the surviving fraction of cells after radiation. This RSI value is then used to individualize the α parameter in the LQ model, which represents linear radiosensitivity (Figure 1). The GARD is computed using the formula: GARD = nd (α + βd), with n being the number of fractions (typically 1); d the dose per fraction; and β is often set as 0.05/Gy^2^. This results in a patient-specific estimate of the cytotoxic effect of radiation, enabling precision radiation therapy that matches dose to tumor biology.

The RSI signature comprises AR (androgen receptor), JUN (c JUN), STAT1, PRKCB (PKCβ), RELA (NF κB subunit RelA), ABL1, SUMO1, PAK2, HDAC1, and IRF1—a set of 10 genes central to transcriptional regulation, stress responses, and signaling pathways [12]. AR, JUN, STAT1, and RELA are transcription factors that orchestrate gene expression programs in response to hormonal signals, cytokines, and inflammatory stimuli, making them key regulators of cellular response to DNA damage. ABL1 and PAK2 are kinases linking cytoskeletal dynamics and cell-cycle progression, while PRKCB mediates signal transduction via the PKC pathway. SUMO1, involved in post translational modification, modulates protein stability and localization. HDAC1 is a chromatin remodeling enzyme critical for epigenetic regulation and DNA repair. Lastly, IRF1 acts as a transcription factor driving interferon stimulated gene expression, promoting cell cycle arrest and apoptosis. The coordinated expression of these genes enables RSI to robustly predict intrinsic radiosensitivity across diverse tumor types.

## 3. Evaluation of GARD in Cancer Studies

In the foundational 2017 study by Scott et al., the GARD model was formally introduced as a clinically applicable framework for personalized radiation dosing [8]. Using gene expression data from over 8000 tumors in the Total Cancer Care (TCC) protocol, the authors calculated GARD values across 20 disease sites and demonstrated wide intratumoral variability in predicted radiotherapeutic effect, even within uniform dose groups. Crucially, GARD was validated as an independent predictor of clinical outcomes in five distinct cohorts—breast (Erasmus and Karolinska), lung, pancreas, and glioblastoma—spanning diverse tumor types and treatment contexts. In multivariable analyses, GARD outperformed both the physical radiation dose and the underlying RSI in predicting outcomes such as distant metastasis-free survival and overall survival. This study established the biological and clinical validity of GARD, laying the groundwork for genomically guided radiotherapy in precision oncology.

A series of studies between 2017 and 2024 expanded the evidence base for GARD across diverse tumor types and clinical contexts (Table 1). Details of the GARD-based radiotherapy dose recommendations and associated clinical outcomes are summarized in Supplementary Table S1. In 2017, Scott JG et al. [8] developed the GARD model using over 8000 tumor samples from the TCC protocol and validated its predictive power across five clinical cohorts. Yuan Z et al. [13] examined 25 PeSCC samples and found standard PORT doses often subtherapeutic, advocating for GARD-guided escalation above 66 Gy. In 2021, Scott JG et al. [9] conducted a pooled pan-cancer analysis (*n* = 1615) across 11 cohorts, showing GARD’s superiority over physical dose in predicting recurrence and survival, while Yang G et al. [14] found that high-risk sarcoma patients required elevated biological effective radiation dose based on GARD. That same year, Scott JG [15] applied GARD to NSCLC cohorts, explaining RTOG 0617 failures and supporting personalized dose modeling. In 2022, Nolan B et al. [16] used RNA-seq to contrast RSI/GARD between tumor and normal tissues in breast and prostate cancers, suggesting tailored dosing. Ho E et al. [17] demonstrated GARD’s prognostic value in HPV+ OPSCC, supporting virtual dose de-escalation trials. Chiang CL [10] validated GARD ≥ 45 as a predictor of locoregional control in NPC, while Naghavi AO [18] integrated GARD with radiomics for targeting resistant habitats in high-grade STS. Kaida A [19] leveraged TCGA data to stratify HNSC tumors by HPV status and radiosensitivity.

In the 2019 study by Yuan et al., the authors assessed radiosensitivity and genomic-adjusted radiation dose (GARD) in penile squamous cell carcinoma (PeSCC), a rare but aggressive malignancy often treated with postoperative radiation therapy (PORT) [13]. Transcriptomic profiling of 25 tumors revealed that most PeSCC lesions were intrinsically radioresistant, with only 16% classified as radiosensitive based on the radiosensitivity index (RSI). GARD modeling demonstrated that the standard PORT dose of 50 Gy provided sufficient therapeutic effect in only 52% of cases, while increasing the dose to 66 Gy improved predicted benefit to 84%. A clinical review of 34 patients supported these findings, with 41% experiencing locoregional recurrence, often within six months of PORT. These results highlight the inadequacy of uniform radiation dosing in PeSCC and underscore the potential of GARD to guide personalized RT strategies that align with tumor biology.

Building on these observations, the study proposed 66 Gy as a more biologically effective dose for the majority of PeSCC patients, particularly those with high RSI and low baseline GARD scores. By recalculating GARD at incrementally higher doses, the authors demonstrated that 66 Gy would shift a substantial proportion of tumors—previously undertreated at 50 Gy—into a therapeutically effective range. This individualized adjustment reflects the core principle of GARD-guided therapy: tailoring dose escalation not uniformly, but selectively based on genomic radiosensitivity. Such modeling provides a rational framework for optimizing therapeutic ratio, offering a potential path forward for dose intensification in patients predicted to benefit from higher radiation exposure, while avoiding unnecessary toxicity in radiosensitive cases. The 66 Gy threshold thus represents an early, data-driven benchmark for biologically guided dose personalization in PeSCC.

A 2019 study by Ahmed et al. investigated the clinical utility of GARD in personalizing adjuvant radiotherapy for triple-negative breast cancer (TNBC) [20]. Using two independent cohorts (*n* = 58 and *n* = 55), the authors demonstrated that GARD was significantly associated with local control: patients with GARD above predefined thresholds (≥23.2 in cohort 1; ≥21 in cohort 2) experienced markedly improved 5-year local control rates (79–96%) compared to those with lower GARD values (55–71%). Modeling individualized dose prescriptions revealed that approximately 91% of TNBC patients would require escalated doses up to 70 Gy to reach the optimal GARD threshold, underscoring the inadequacy of uniform dosing in this aggressive subtype. These findings support the feasibility of GARD as a biologically driven tool to tailor RT dose for TNBC patients.

In the landmark 2021 study published in *The Lancet Oncology*, Scott et al. conducted a pooled pan-cancer analysis to evaluate the prognostic value of the genomic-adjusted radiation dose (GARD) across 1615 patients from 11 cohorts encompassing seven different cancer types [9]. By integrating tumor-specific radiosensitivity index (RSI) with clinical radiation dose data, the authors demonstrated that GARD significantly outperformed physical radiation dose in predicting recurrence and overall survival outcomes. The study revealed that patients receiving the same physical dose exhibited a broad distribution of GARD values, emphasizing biological variability in tumor response. GARD was found to be a continuous predictor of clinical benefit, with higher GARD values consistently associated with improved outcomes. This analysis provided strong retrospective validation of GARD as a pan-cancer biomarker of radiotherapeutic effect, reinforcing its role as a key component in precision radiotherapy planning.

Yang et al. (2021) applied the GARD framework to evaluate intertumoral radiosensitivity in soft tissue sarcomas (STS) [14], a heterogeneous and historically radioresistant cancer type. By analyzing 217 genomically profiled sarcomas using the radiosensitivity index (RSI), the authors identified distinct molecular subtypes, including homologous recombination repair (HRR)-deficient tumors, which exhibited significantly higher predicted biological effective dose requirements for optimal local control. Retrospective analysis of 399 clinically treated STS patients revealed that standard radiation dosing was insufficient for the HRR-deficient subset, supporting the need for biologically guided dose escalation. These findings demonstrated the utility of GARD to uncover actionable heterogeneity within sarcoma subtypes and suggested that integrating genomic information can guide more effective, stratified radiotherapy strategies in STS management.

Nolan et al. (2022) demonstrated the feasibility of applying the genomic-adjusted radiation dose (GARD) model using RNA-seq data from both tumor and adjacent normal tissues in breast and prostate cancers [16]. By calculating the radiosensitivity index (RSI) and GARD from transcriptomic data, they found significant inter- and intrapatient variability in predicted radiation sensitivity. For a luminal B breast cancer patient, tumor tissues showed markedly higher GARD values than matched normal tissues, supporting the concept of tumor-selective radiation dosing. In prostate cancer, large interpatient GARD variability suggested that standard doses may under- or overtreat patients based on tumor biology. This study underscores the potential of RNA-seq-derived GARD to inform personalized radiotherapy strategies that optimize efficacy while minimizing toxicity.

Ho et al. (2023) investigated the utility of GARD in HPV-positive oropharyngeal squamous cell carcinoma (HPV+ OPSCC) using transcriptomic data from 234 patients profiled with Affymetrix Clariom D microarrays [17]. By integrating GARD with clinical variables, the authors developed a clinicogenomic model that significantly outperformed the NRG clinical nomogram in predicting overall survival. High-GARD tumors were associated with improved outcomes, and a virtual trial simulation suggested that approximately 36% of patients could be eligible for GARD-informed radiation dose de-escalation without compromising therapeutic efficacy. These findings support the use of GARD not only as a prognostic biomarker but also as a potential tool for safely reducing treatment intensity in favorable-risk HPV+ OPSCC patients, thereby minimizing toxicity while maintaining oncologic control.

Chiang et al. (2024) assessed the prognostic value of GARD in nasopharyngeal carcinoma (NPC) using data from 92 patients enrolled in a Phase III randomized trial (NCT00379262) [10]. The study employed gene expression data to calculate both the radiosensitivity index (RSI) and GARD, aiming to stratify patients by predicted radiation response. A GARD threshold of ≥45 was identified as a significant predictor of improved locoregional control, independent of traditional clinical variables. Notably, GARD outperformed both physical dose and RSI alone in multivariate models, supporting its role as a biologically informed metric of radiotherapy efficacy. These findings provide strong clinical evidence that GARD may be used to personalize radiation dose in NPC, identifying patients who may benefit from dose escalation and sparing those for whom standard treatment is sufficient.

A 2024 study by Huang et al. validated the GRAD model in a Chinese cohort of 64 patients with locally advanced rectal cancer (LARC) undergoing neoadjuvant chemoradiotherapy [21]. Using gene expression profiling to calculate the radiosensitivity index (RSI) and GARD, the study demonstrated that higher GARD scores (≥17) were significantly associated with improved disease-free survival and lower neoadjuvant rectal (NAR) scores, indicating better treatment response. Notably, only 17% of patients had personalized GARD-predicted doses (pGRT) within the standard guideline range of 45–50 Gy, with the remainder either under- or over-dosed. These findings underscore the biological heterogeneity among patients and support the clinical utility of GARD in guiding individualized radiotherapy dosing strategies in rectal cancer care.

Naghavi et al. (2024) reported the early results of the Phase II HEAT (Habitat Escalated Adaptive Therapy) trial [18], which prospectively evaluated the integration of GARD and radiomic habitat-guided dose escalation in high-grade soft tissue sarcomas (STS). The study leveraged multiparametric MRI to define intratumoral habitats characterized by distinct biological features such as hypoxia and cellularity, with biopsy-confirmed genomic profiling to calculate GARD values for each region. Radiation therapy was delivered using simultaneous integrated boost (SIB) techniques at 50, 60, or 70 Gy based on habitat-specific radiosensitivity, achieving isotoxic escalation while preserving normal tissue constraints. This trial demonstrated the feasibility and safety of combining radiomics and GARD to individualize radiotherapy in real-time and laid the groundwork for future precision oncology approaches that tailor dose not just to the patient, but to the spatial heterogeneity within the tumor.

Conventional fixed-fractionation radiotherapy regimens have the advantage of extensive clinical validation, standardization, and simplicity, as evidenced by landmark trials across tumor types. For example, in stage III lung cancer RTOG 0617, a uniform dose-escalation (74 Gy vs. 60 Gy) was tested in all patients; the high-dose arm was unexpectedly inferior and closed early for futility, reaffirming the 60 Gy standard established since the 1980s [22]. In prostate cancer, the Phase III CHHiP trial showed that a hypofractionated schedule (60 Gy in 20 fractions) was non-inferior to the conventional 74 Gy/37 fractions with no increase in late toxicities [23], leading moderate hypofractionation to be adopted as a new standard of care [24]. Similarly, the UK START trials in early breast cancer demonstrated that appropriately dosed hypofractionation (e.g., 40 Gy in 15 fractions over 3 weeks) achieves equivalent 10-year tumor control and even reduced some late normal-tissue effects compared to the historical 5-week regimen [25]. These fixed regimens benefit from long-term follow-up confirming efficacy and safety, and their one-size-fits-all dosing is logistically straightforward—shorter courses like CHHiP and START also improved patient convenience and resource utilization [26]. By contrast, GARD-guided dosing requires tumor genomic profiling to tailor the radiation dose, using a 10-gene expression radiosensitivity index derived from biopsy tissue [8]. While this promises a biologically personalized approach, GARD remains unproven in large prospective trials (to date no Phase III biomarker-driven trial has validated its clinical benefit, and only a Phase II is in progress) [1]. Moreover, basing dose on a single genomic snapshot risks overlooking intratumoral heterogeneity—spatial clonal variations and tumor microenvironment factors that a limited gene signature might miss—underscoring technical and biological challenges in applying GARD broadly [27]. In summary, conventional fractionation schemes are backed by decades of robust trial data and are simple to implement, whereas GARD-based dose adjustment, though conceptually appealing, faces hurdles of requiring complex genomic assays, lacking extensive prospective validation, and potential insensitivity to tumor heterogeneity, which must be addressed before it can rival the proven reliability of standard fractionation [1,25]. A detailed comparison between GARD-guided radiotherapy and conventional dose fractionation is provided in Supplementary Table S2.

## 4. GARD-Personalized Dosing in Clinical Trial

A new Phase II clinical trial from Moffitt Cancer Center represents a critical step forward in prospectively validating GARD-personalized radiotherapy in HPV-positive oropharyngeal squamous cell carcinoma (OPSCC), using a noninferiority/superiority hybrid design [28]. Building on prior retrospective findings that GARD outperforms traditional clinical nomograms in predicting survival, this trial is designed to determine whether individualized dose prescriptions based on GARD can maintain clinical efficacy while reducing toxicity. The study employs a Bayesian framework with interim analyses and uses 3-month PET response as the primary endpoint, offering a biologically informed alternative to empirical de-escalation strategies that have failed in prior trials. If successful, the trial may establish GARD not only as a prognostic biomarker but also as a prospective clinical tool for stratified radiation dosing in head and neck cancer.

The study is a single-arm, non-randomized Phase II trial evaluating whether GARD-personalized RT dosing (ranging from 54 to 82 Gy) can maintain non-inferior clinical outcomes compared to historical controls. All patients will receive concurrent platinum-based chemotherapy. The primary endpoint is the metabolic complete response rate assessed by PET/CT three months post-treatment. Secondary endpoints include acute toxicity and 1-year progression-free survival. Eligibility criteria include newly diagnosed, HPV-positive Stage I–III oropharyngeal cancer, with sufficient biopsy tissue available for GARD profiling. Patients with prior head and neck cancers, distant metastases, or poor performance status will be excluded. A Bayesian statistical framework will be used, with historical response rates set at 95% and an effectiveness threshold defined as >90% posterior probability of success. The trial plans to accrue 35 patients, with interim analyses every 10 patients to update priors and assess trial continuation. This design emphasizes feasibility and iterative learning, with the potential to inform a future randomized trial. Even if the primary endpoint is not met, the study is expected to yield valuable data to further refine the clinical utility of GARD in radiation personalization.

## 5. Discussion and Future Directions

Over the past decade, a growing body of evidence has highlighted the potential of the genomic-adjusted radiation dose (GARD) to transform radiotherapy from a one-size-fits-all paradigm to a biologically personalized treatment strategy. Retrospective studies across multiple tumor types—including breast, prostate, head and neck, sarcoma, and rectal cancers—have consistently demonstrated GARD’s superior prognostic and predictive performance compared to physical dose alone. More recent investigations, including the HEAT and Moffitt Phase II trials, mark a shift toward prospective validation of GARD-guided dosing strategies in clinical settings. These studies not only confirm the biological heterogeneity in tumor radiosensitivity but also provide actionable thresholds for optimizing dose intensity. The cumulative findings support the clinical feasibility of using GARD to tailor radiotherapy, especially in tumors where standard dose protocols fall short. As the field evolves, the integration of GARD with radiomics, artificial intelligence, and multiparametric imaging holds promise for real-time, spatially resolved dose adaptation—paving the way for more precise and effective cancer treatment.

GARD offers a promising strategy for biologically guided radiation dosing and personalized radiotherapy. The model’s foundational appeal lies in its ability to translate tumor-specific genomic information into a quantifiable estimate of radiation effect, potentially guiding dose adjustments that improve clinical outcomes. Initial studies demonstrated prognostic value across several tumor types and established GARD’s superiority over uniform physical dose in retrospective analyses. However, concerns have been raised regarding its robustness and clinical validity. The core input to GARD—the radiosensitivity index (RSI) was originally derived from in vitro experiments using the NCI-60 panel of human cancer cell lines, based on their surviving fraction at 2 Gy of radiation [12]. While this provided a reproducible and scalable framework for modeling radiosensitivity, the use of cell lines fails to recapitulate the complexity of the in vivo tumor microenvironment. Critical factors like hypoxia, stromal, and immune interactions, and tumor heterogeneity—which influence radiation response—are not captured in vitro. As a result, RSI may not fully reflect the biological behavior of tumors in patients, potentially limiting its prognostic and predictive utility in clinical settings [29,30]. To improve the predictive accuracy and clinical relevance of the radiosensitivity index (RSI), one promising direction is to expand its derivation and validation beyond the original NCI-60 panel by incorporating larger, more diverse cancer cell line datasets. Modern resources such as the Cancer Cell Line Encyclopedia (CCLE) [31,32,33] and Genomics of Drug Sensitivity in Cancer (GDSC) [34,35] include hundreds of well-characterized cell lines across many tumor types, with available gene expression profiles and radiation sensitivity phenotypes. These broader datasets offer the potential to capture a wider spectrum of radiosensitivity mechanisms, tumor subtypes, and biological heterogeneity, thereby enhancing the generalizability of RSI across different cancers [36,37]. Additionally, integrating data on microenvironmental conditions—such as hypoxia or co-culture with immune/stromal cells—may further refine model accuracy by approximating in vivo tumor contexts.

Another limitation in the current body of evidence supporting GARD is that many published studies to date are retrospective in nature. These analyses often rely on archived gene expression datasets and post hoc associations with clinical outcomes, which may be subject to biases such as selection bias, confounding, and missing data. Retrospective designs also limit the ability to assess the real-time feasibility and clinical utility of incorporating GARD into treatment decision-making. Without prospective validation—ideally through interventional trials that randomize patients based on GARD-informed dose strategies—it remains unclear whether modifying radiation prescriptions according to GARD will translate into improved clinical outcomes. Thus, while retrospective studies have been valuable in hypothesis generation and exploratory validation, prospective clinical trials are essential to establish GARD as a robust and actionable biomarker for radiation personalization. A notable prospective effort to validate GARD in a clinical setting is the HEAT trial (NCT05301283) [18], a Phase II single-arm study designed to evaluate the feasibility and effectiveness of radiomic habitat-directed, GARD-optimized radiation therapy in patients with high-grade, resectable soft tissue sarcomas. This trial pioneers the integration of GARD with advanced imaging modalities to personalize radiation dose escalation within tumors, using multiparametric MRI to define distinct intratumoral habitats with varying degrees of hypoxia and cellularity. Each habitat receives isotoxic doses of 50, 60, or 70 Gy using simultaneous integrated boost (SIB) techniques. The HEAT trial represents a step in translating GARD from retrospective analysis to prospective clinical practice, offering a model for how genomically informed radiation dosing can be implemented in real-time to improve tumor response while minimizing normal tissue toxicity.

Due to spatial heterogeneity within tumors, a single biopsy may not capture the full genomic diversity across the irradiated volume [38,39,40]. Moreover, tumors evolve dynamically during the course of therapy, and radiation itself can induce clonal selection or promote mutational changes, thereby altering radiosensitivity profiles over time. Such treatment-induced heterogeneity challenges the validity of applying a static, pre-treatment RSI to guide dose throughout the course of radiation. These concerns are compounded by the fact that many studies have not confirmed whether the sampled tissue overlaps anatomically with the irradiated tumor, potentially misrepresenting the biological characteristics used in GARD computation. As such, the temporal and spatial mismatch between biopsy and treatment target may undermine the clinical utility of RSI and GARD as robust predictive tools in precision radiotherapy.

An important perspective on the clinical implications of GARD was articulated by Kaidar-Person, Poortmans, and Salgado in their commentary accompanying the 2021 Lancet Oncology publication [41]. They highlighted that while the development of GARD represents a major advancement in bridging genomics and radiotherapy, radiotherapy as a field has lagged behind systemic therapy in embracing personalized medicine [9]. A commentary published alongside the 2021 *Lancet Oncology* study by Scott et al. further emphasized the significance of GARD as a pioneering approach for individualizing radiotherapy based on biological effect rather than physical dose alone [42]. The authors commended the conceptual innovation of GARD but emphasized the need for a clearer understanding of its biological underpinnings. Specifically, they questioned whether the ten-gene RSI signature reflects causal drivers of radiosensitivity or simply serves as a surrogate readout of broader genomic networks. In response, Scott and colleagues clarified that the RSI signature is not necessarily mechanistically deterministic but instead functions as a network-based biomarker that captures emergent properties of tumor radiosensitivity. This view positions the genes as indicators of the overall system state rather than as direct effectors of radiation response. The commentary authors also underscored the importance of integrating GARD into prospective clinical trials under frameworks proposed by the U.S. National Cancer Institute, which include assay standardization, analytic validity, and demonstration of clinical utility. This feedback reinforces the importance of continued refinement and validation of GARD in real-world clinical workflows before widespread adoption.

A recent review examining the role of artificial intelligence (AI) in lung cancer treatment underscores AI’s transformative promise for precision-guided radiotherapy dose adjustment [43]. The authors highlight that AI algorithms—ranging from machine learning to deep learning and natural language processing—can analyze high-dimensional data including imaging, genomic profiles, and clinical variables to predict tumor radiosensitivity, treatment response, and prognosis in lung cancer patients. This multimodal analytical framework can support biologically informed dosing strategies, enabling personalized tailoring of radiation dose based on predicted tumor behavior rather than solely on empirical protocols. By integrating imaging-derived radiomic features with gene expression and clinical metrics, AI models can suggest individualized biologically effective doses and identify patients likely to benefit from dose escalation or de-escalation. These capabilities align closely with the GARD paradigm and suggest a scalable AI-augmented pathway toward optimizing radiotherapy dose prescription in lung cancer and other tumors with heterogeneous radiosensitivity.

## 6. Conclusions

The development and validation of the genomically adjusted radiation dose (GARD) framework offer a promising step toward more personalized radiation therapy. By incorporating tumor-specific radiosensitivity into dose planning, GARD introduces a biologically informed approach to radiotherapy that may enhance treatment precision. Early clinical studies, including a Phase II trial in HPV-positive oropharyngeal cancer, suggest the feasibility and potential value of biologically guided dose adaptation. As further research evaluates its clinical utility and integration into routine practice, GARD may contribute to improving therapeutic outcomes while managing toxicity in selected patient populations.

## Figures and Tables

**Table 1:** Studies to evaluate GARD.

Year	First Author	Country *	Sample Size	Cancer Type(s)	Summary	DOI
2017	Scott JG [8]	USA; The Netherlands	538 patients (5 cohorts)	Multi-cancer (20 tumor types; validated in breast, lung, pancreas, glioblastoma)	Developed GARD model; analyzed > 8000 tumors (TCC protocol); validated GARD as outcome predictor in 5 clinical cohorts (breast, lung, pancreas, glioblastoma).	10.1016/S1470-2045(16)30648-9
2019	Yuan Z [13]	USA	34 patients	Penile squamous cell carcinoma (PeSCC)	25 PeSCC samples analyzed for RSI and GARD; 34 patients reviewed; suggested standard PORT dose (50 Gy) often subtherapeutic, with GARD-based RT > 66 Gy more effective.	10.1016/j.rpor.2019.09.006
2019	Ahmed KA [20]	USA; The Netherlands; France	113 TNBC patients (Cohort 1: 58; Cohort 2: 55)	Triple-negative breast cancer (TNBC)	GARD was significantly associated with local control in both cohorts. Patients with GARD < 21 had worse 5-year LC (71% vs. 96%). Dose modeling suggested that 91% of patients would require ≥ 70 Gy to optimize GARD, indicating standard dosing may undertreat most TNBC cases.	10.1016/j.ebiom.2019.08.019
2021	Scott JG [9]	USA	1615 patients (pooled from 11 cohorts)	Multi-cancer (breast, head and neck, NSCLC, pancreas, endometrial, melanoma, glioma)	Pooled pan-cancer analysis of 11 cohorts (*n* = 1615, 7 cancer types); GARD associated with improved time to recurrence and OS; outperformed physical RT dose.	10.1016/S1470-2045(21)00347-8
2021	Yang G [14]	USA	399 patients	Soft-tissue sarcoma (STS)	217 genomically profiled sarcomas and 399 treated STS patients; HRR subset required significantly higher biological effective radiation dose for optimized RT outcomes.	10.1016/j.tranon.2021.101165
2021	Scott JG [15]	USA	1747 patients (genomic cohort) + 60 patients (validation cohort)	Non-small cell lung cancer (NSCLC)	NSCLC patients from two cohorts used to validate a personalized RT dosing model; GARD explains failure of RTOG 0617; proposes GARD-based dose optimization.	10.1016/j.jtho.2020.11.008
2022	Nolan B [16]	Ireland	15 patients (1 breast; 14 prostate)	Breast (luminal B) and prostate cancer	RNA-seq data from 1 breast cancer and 14 prostate cancer patients; explored tumor vs. normal RSI and GARD values to propose individualized dosing strategies.	10.1016/j.ctro.2022.08.002
2023	Ho E [17]	USA; Italy; The Netherlands; Germany	191 patients	Oropharyngeal squamous cell carcinoma (HPV-positive)	234 HPV+ OPSCC samples (Affymetrix Clariom D); GARD predicted OS and outperformed NRG nomogram; virtual trial suggests a GARD-based dose de-escalation strategy.	10.1101/2023.09.14.23295538
2024	Chiang CL [10]	Hong Kong (China); USA	92 patients	Nasopharyngeal carcinoma (NPC)	92 NPC patients from a Phase III trial (NCT00379262); evaluated GARD and RxRSI to stratify patients by radiosensitivity; a GARD threshold of 45 associated with improved locoregional control.	10.1016/j.radonc.2024.110287
2024	Naghavi AO [18]	USA	Up to 43 patients (Phase II trial, planned)	High-grade soft tissue sarcoma (STS)	Phase II HEAT trial in high-grade STS using radiomics + GARD to boost RT in resistant tumor habitats; pre-treatment mpMRI and biopsies used for RT planning; NCT05301283.	10.1186/s12885-024-12151-7
2024	Huang X [21]	China	64 patients (plus external validation from GSE40492)	Locally advanced rectal cancer (LARC)	Validated GARD in Chinese LARC patients; GARD > 17 predicted better DFS and response. Only 17% had doses within standard 45–50 Gy, highlighting need for personalized RT.	10.1038/s41598-024-72818-w
2024	Kaida A [19]	Japan	~110 patients (TCGA HNSC cohort)	Head and neck squamous cell carcinoma (HNSC, stratified by p16/HPV status)	Used TCGA data to estimate GARD in HNSC; p16+ tumors had higher radiosensitivity and therapeutic GARD; supports stratified RT by HPV status.	10.1667/RADE-24-00066.1

* Each study’s country corresponds to the primary research institutions.

## Supplementary Materials

The following supporting information can be downloaded at: https://www.mdpi.com/article/10.3390/cancers17162650/s1, Table S1: Suggested GARD-based doses and clinical outcomes from studies to evaluate GARD; Table S2: Comparison of GARD versus Conventional Radiotherapy Dose Fractionation.
