# Supplementary material for: Narrative Review of the Use of Genomic-Adjusted Radiation Dose (GARD) in Radiotherapy
Source: Cancers (Basel). 2025 Aug 14;17(16):2650. doi: 10.3390/cancers17162650 (PMC12384496; doi:10.3390/cancers17162650)
Supplement: Supplementary file 1 [file cancers-17-02650-s001.zip › cancers-3757965-supplementary.pdf]

Table S1. Suggested GARD-based doses and clinical outcomes from studies to evaluate GARD.

Year	First Author	Suggested GARD-based Dose	Clinical Outcome
2017	Scott JG[8]	GARD modeling indicated that the same physical RT dose (e.g., 50–70 Gy) could yield markedly different biological effects depending on tumor genomics.	Validated GARD as an independent predictor of outcome across multiple cohorts. Patients with high GARD had significantly better 5-year distant metastasis–free survival than those with low GARD.
2019	Yuan Z[13]	Suggested escalating post-operative RT in penile SCC from the standard ~50 Gy to ~66 Gy. GARD modeling indicated ~66 Gy would yield therapeutic benefit in ~84% of cases (vs only ~52% benefit at 50 Gy).	Standard 50 Gy was often subtherapeutic (41% locoregional recurrence rate); a higher GARD-derived dose (~66 Gy) was predicted to markedly improve locoregional control.
2019	Ahmed KA[20]	Estimated that ~91% of triple-negative breast cancer (TNBC) patients would require escalated RT doses ≥70 Gy to achieve optimal GARD (≥21), suggesting that standard dosing may undertreat the majority of TNBC cases.	Demonstrated that patients with GARD ≥21 had significantly improved 5-year local control (96%) compared to those with GARD <21 (71%), supporting GARD-based dose personalization to enhance outcomes in this aggressive breast cancer subtype.
2021	Scott JG[9]	Proposed using GARD to personalize RT prescriptions instead of one-size-fits-all dosing (integrating tumor genomics into dose selection). Suggesting a shift from fixed RT dosing (e.g., 50–70 Gy) to personalized GARD-based dosing.	In a pooled analysis of 7 cancer types, GARD was significantly associated with longer time to recurrence and overall survival, whereas physical RT dose showed no association. Higher GARD predicted greater benefit from RT, outperforming actual dose as a predictor of outcome.
2021	Yang G[14]	Calculated that highly radioresistant soft-tissue sarcomas would require an extremely high dose (~97 Gy BED, α/β=3.29) to achieve an optimal GARD and treatment effect.	Identified a "highly radioresistant" (HRR) subset of sarcomas with much poorer response to standard RT – only ~2.4% pathologic response and 76.5% 5-year local control (vs 19.4% and 90.8% in others). This indicates standard doses are insufficient for HRR tumors, necessitating dose intensification for better outcomes.
2021	Scott JG[15]	Stratified NSCLC patients into three groups by GARD-based needed dose: (1) radiosensitive tumors – adequately treated at ~60 Gy (standard dose), (2) intermediate – may benefit from moderate dose escalation (~70–75 Gy), (3) resistant – likely require >74 Gy to control disease.	GARD-based modeling explained the failure of uniform high-dose escalation in RTOG 0617. It showed that only a subset of patients benefit from higher doses. Personalizing dose by GARD could improve local control and survival, whereas empiric 74 Gy for all led to no OS benefit.
2022	Nolan B[16]	Using RNA-seq RSI data, found that about half of analyzed prostate tumors would require a higher total dose than the standard 72 Gy (i.e. >72 Gy) to reach a "high" GARD value (high therapeutic effect). Tumor-vs-normal RSI comparisons were explored to guide individualized dosing.	This exploratory study showed clear differences in RSI/GARD between tumor and normal tissue for some patients. It suggests that combining GARD with transcriptomics could inform personalized RT dosing – for example, many prostate cancer patients might need dose escalation beyond 72 Gy to achieve

			optimal tumor control. No direct clinical outcomes were reported; study demonstrates potential for personalized dosing to improve control.
2023	Ho E[17]	Advised against uniform dose de-escalation in HPV-positive oropharyngeal cancer. A virtual trial showed that lowering RT dose for all such patients would worsen survival. Instead, two GARD-guided strategies were proposed: selectively de-escalate RT only in patients whose tumors have high GARD (genomically radiosensitive), while maintaining standard dose in others.	GARD strongly predicted overall survival in HPV+ OPSCC. Each unit increase in GARD corresponded to a 5% reduction in hazard of death (HR ≈ 0.95), and GARD outperformed an NRG clinical nomogram for OS prediction. Patients with GARD ≥64.2 (high radiosensitivity) had significantly improved OS, supporting the selective de-intensification approach to preserve outcomes.
2024	Chiang CL[10]	Identified three nasopharyngeal carcinoma subgroups based on GARD ≥45 (threshold for good control): ~64% of tumors were so radiosensitive that GARD ≥45 was reached with <66 Gy; ~22% needed a dose in the 66–74 Gy range; ~14% were radioresistant, requiring >74 Gy to achieve GARD ≥45.	GARD was independently prognostic in NPC. Tumors achieving GARD ≥45 had significantly higher locoregional failure-free survival (p=0.008). This suggests patients with lower GARD (at standard dose) might benefit from dose escalation, whereas many others could be adequately treated with lower-than-standard doses.
2024	Naghavi AO[18]	(HEAT Trial, in progress) Implementing GARD- and radiomics-guided dose boosts in high-grade STS. Standard neoadjuvant RT is 50 Gy; in this trial, resistant tumor "habitats" (mpMRI-defined) receive boosted doses (e.g. 60 Gy to intermediate-risk regions and 70 Gy to the most radioresistant regions) while the rest of the tumor gets standard dose.	Outcome pending (ongoing Phase II trial). The trial's goal is to significantly improve pathological complete response rates – projected to increase ≥3-fold (from ~8% with standard 50 Gy to ~24% with GARD-optimized boosts). Success would provide first clinical evidence that GARD-personalized dose escalation improves outcomes in sarcoma.
2024	Huang X[21]	Estimated that only 17% of LARC patients had personalized GARD-predicted doses (pGRT) within the guideline range of 45–50 Gy; 45% were below and 38% above this range, indicating significant mismatch between standard RT doses and genomic needs.	Patients with GARD ≥17 had significantly lower NAR scores and better outcomes, including 72% 5-year DFS in external validation, supporting the need for GARD-based personalization to improve radiotherapy efficacy in rectal cancer.
2024	Kaida A[19]	Proposed stratifying RT dose by HPV status in head & neck cancer. Using TCGA data, the study assumed HPV/p16-positive patients could be treated with 60 Gy vs 70 Gy for p16-negative patients (reflecting de-escalation for radiosensitive, HPV+ tumors).	Confirmed that p16-positive (HPV+) HNSCC tumors are intrinsically more radiosensitive. RSI/GARD analysis showed HPV+ cases have a higher therapeutic effect from a given dose than HPV– cases. This supports dose de-intensification in HPV+ patients (who achieve high GARD with lower dose) while keeping full dose for HPV-negative disease.

Table S2. Comparison of GARD versus Conventional Radiotherapy Dose Fractionation.

Aspect	GARD (Genomic-Adjusted Radiation Dose)	Conventional Radiotherapy Dose Fractionation
Personalization	Tailors dose based on tumor-specific radiosensitivity using a 10-gene expression profile [8,12].	One-size-fits-all dosing; not personalized [22,24].

Aspect	GARD (Genomic-Adjusted Radiation Dose)	Conventional Radiotherapy Dose Fractionation
Scientific Rationale	Biologically informed; integrates genomic data into the LQ model for precision dosing [8,9,10].	Based on empirical outcomes from historical and large randomized trials [24–26].
Validation	Limited: No phase III trial has yet confirmed clinical benefit; one phase II ongoing [18].	Extensive: Backed by decades of randomized phase III trials across cancer types [22–26].
Implementation Complexity	Requires tumor biopsy, RNA expression profiling, and computational modeling [8,12,16].	Simple to implement; no biomarker testing required [22–26].
Evidence for Outcome Prediction	Promising retrospective results; GARD correlated with clinical outcomes and RT benefit [8–10,13–21].	Strong evidence for tumor control, toxicity, and survival from large-scale trials [22–26].
Tumor Heterogeneity	May miss spatial/temporal heterogeneity; snapshot from one biopsy may be insufficient [27,38–40].	Less affected by molecular heterogeneity since uniform dose is applied empirically [27].
Adaptability	Offers potential to escalate or de-escalate RT dose for individual tumors [13,14,17,18].	Dose schemes are fixed and not dynamically adjustable per patient biology [1, 25].
Clinical Adoption	Experimental; not standard of care [1,9,18].	Widely adopted as standard of care globally [22–26].
Logistical Burden	Higher: requires genomic infrastructure, added cost, and turnaround time [8,16,27].	Lower: protocols already integrated into clinical workflows [22–26].
Patient Convenience	Could reduce overtreatment or spare normal tissue in some patients [17,21].	Hypofractionated schedules (e.g., START [25], CHHiP [23]) already improve convenience.
